# Real-Time Dynamics of Ca^2+^, Caspase-3/7, and Morphological Changes in Retinal Ganglion Cell Apoptosis under Elevated Pressure

**DOI:** 10.1371/journal.pone.0013437

**Published:** 2010-10-18

**Authors:** Jae Kyoo Lee, Siyuan Lu, Anupam Madhukar

**Affiliations:** 1 Department of Biomedical Engineering, University of Southern California, Los Angles, California, United States of America; 2 Department of Physics, University of Southern California, Los Angles, California, United States of America; 3 Department of Ophthalmology, University of Southern California, Los Angles, California, United States of America; 4 Department of Materials Science, University of Southern California, Los Angles, California, United States of America; Universidade Federal do Rio de Janeiro, Brazil

## Abstract

Quantitative information on the dynamics of multiple molecular processes in individual live cells under controlled stress is central to the understanding of the cell behavior of interest and the establishment of reliable models. Here, the dynamics of the apoptosis regulator intracellular Ca^2+^, apoptosis effector caspase-3/7, and morphological changes, as well as temporal correlation between them at the single cell level, are examined in retinal gangling cell line (differentiated RGC-5 cells) undergoing apoptosis at elevated hydrostatic pressure using a custom-designed imaging platform that allows long-term real-time simultaneous imaging of morphological and molecular-level physiological changes in large numbers of live cells (beyond the field-of-view of typical microscopy) under controlled hydrostatic pressure. This examination revealed intracellular Ca^2+^ elevation with transient single or multiple peaks of less than 0.5 hour duration appearing at the early stages (typically less than 5 hours after the onset of 100 mmHg pressure) followed by gradual caspase-3/7 activation at late stages (typically later than 5 hours). The data reveal a strong temporal correlation between the Ca^2+^ peak occurrence and morphological changes of neurite retraction and cell body shrinkage. This suggests that Ca^2+^ elevation, through its impact on ion channel activity and water efflux, is likely responsible for the onset of apoptotic morphological changes. Moreover, the data show a significant cell-to-cell variation in the onset of caspase-3/7 activation, an inevitable consequence of the stochastic nature of the underlying biochemical reactions not captured by conventional assays based on population-averaged cellular responses. This real-time imaging study provides, for the first time, statistically significant data on simultaneous multiple molecular level changes to enable refinements and testing of models of the dynamics of mitochondria-mediated apoptosis. Further, the platform developed and the approach has direct significance to the study of a variety of signaling pathway phenomena.

## Introduction

Apoptosis, a term first coined by Kerr et al. in 1972 [1], refers specifically to an energy-dependent, genetically controlled cell suicide process by which unneeded or damaged single cells self-destruct to keep the homeostasis of function and structure of a tissue or organism. The failure of regulation of apoptosis leads to abnormalities such as developmental defects, cancer, autoimmune diseases, and neurodegeneration [2]. Studies of apoptosis have typically focused on identifying the involved molecular components and major pathways of apoptosis (intrinsic and extrinsic pathways, or mitochondria-mediated and non-mitochondria-mediated) pathways [2]–[4]. However, little is known about the real-time dynamics of even the identified prominent molecular processes involved in the major apoptotic pathways. Beyond identifying the pathway of apoptosis [2]–[4] and theoretical modeling [5], [6] to shed light on the dynamics and mechanistic relationship of molecular processes contributing to apoptosis, recently efforts have begun to measure the dynamics of the molecular level processes occurring in apoptosis [7], [8]. However, the dominant methodology for collecting molecular information on apoptosis continues to rely on the averaging of the biomarker concentration over a population of typically thousands to millions of fixed or lysed cells at limited particular temporal stages. While this paradigm is very useful for checking the involvement of a particular cellular process to a disease-inducing stress, it falls short in two respects: (1) it does not allow observation of transient cellular processes, and (2) given the large cell-to-cell variation in the response, such population-averaged measurements mask the true nature of time evolution of cellular processes. Moreover, even if more than a single biomarker is observed, it does not allow examination of the time-correlation between molecular processes in individual cells. To overcome these short-comings, we have developed an imaging system that enables real-time quantitative measurements of (a) the changes in the cell morphological features and (b) fluorescence intensities from multiple biomarkers which probe multiple cellular processes involved in apoptosis as a function of time in a large number of individual live cells (in areas beyond the field of view of a typical optical microscope) via time-multiplexing in the same experimental run. Such real-time measurements also make it possible to compare and correlate the time-evolution of different cellular processes in individual cells. Furthermore, since a large number of cells are imaged, the statistical cell-to-cell variation can be revealed and quantified.

Here we report on a study of the dynamics of apoptosis of retinal ganglion cells (RGC). In this study, the RGC apoptosis is induced via elevated hydrostatic pressure ∼100 mmHg comparable to the typical intraocular pressure (IOP) of acute angle-closure glaucoma [9], [10]. While in-vivo studies revealed RGC apoptosis in response to elevated pressure [11], [12], recently established retinal ganglion cell line RGC-5 [13] enables the investigations of the molecular level processes in RGC apoptosis under elevated pressure. Agar et al. studied the apoptosis of undifferentiated RGC-5 cells using the apoptotic marker TUNEL and Phosphatidyl Serine [10]. Liu et al. [14] and Ju et al. [15] reported the increase of oxidative stress and mitochondria fission during RGC-5 apoptosis under pressure. More recent results from the same group demonstrated the release of cytochrome c from mitochondria and caspase-3 activation in differentiated RGC-5 cells measured individually after three days of undergoing 30 mmHg pressure [16]. These results of mitochondria fission and cytochrome c release from the mitochondria by Ju et al. suggest that RGC apoptosis under elevated pressure is governed by mitochondria-mediated pathway.

In the mitochondria-mediated apoptosis, intracellular Ca^2+^ redistribution between cytosol and endoplasmic reticulum (ER) due to efflux from the ER triggered by the mitochondria-released cytochrome c binding in the IP3 receptor (IP3R) was shown [3] by Boehning et al. to play the role of regulating apoptosis [17]. Another key player in apoptosis is the enzymatic protease caspase-3 that cleaves cytoskeleton proteins, finally dismantling the cell structure [18]. Boehning et al. showed also that Ca^2+^ elevation is upstream of caspase-3 activation [3]. However, Assefa et al. [19] reported that Ca^2+^ elevation was inhibited in apoptotic DT40 cells expressing caspase-3-non-cleavable mutant IP3 receptor type I (IP3R1) and the Ca^2+^ elevation was blocked in apoptotic DT40 cells with wild type IP3 receptor by caspase-3 inhibitor, suggesting Ca^2+^ elevation occurs as a consequence of the cleavage of IP3R1 by the activated caspase-3. The molecular processes influencing apoptotic morphological changes such as cell body shrinkage is another unsettled issue in apoptosis. Dismantling of the cytoskeleton proteins that support cell structure is widely held to be a late stage process brought about by the activation of caspase-3 (hence dubbed “effector”) [20] but Maeno et al. [21] attribute observed early cell body shrinkage to osmotic pressure induced by water efflux owing to ion channel activities.

In the present investigation, the dynamics of intracellular Ca^2+^, caspase-3/7, and morphological changes of cell body shrinkage and neurite retraction are measured by simultaneously imaging these processes in a large number of individual live RGCs undergoing apoptosis under controlled elevated pressure over prolonged times. Such measurements provide, for the first time, a quantitative data base for the development of models of a mitochondria-mediated apoptosis process. This is a needed first step towards models relevant to disease onset, prevention, monitoring, and treatment of neuropathy in RGCs.

## Materials and Methods

In this study, retinal ganglion cell line RGC-5 [13] was used to investigate pressure-induced apoptosis. So far RGC-5 is the only available robust RGC cell line and has many similarities to primary RGCs [13], [22]. Thus it is well suited as a preliminary model for our study while also serving as a vehicle for the development of real-time imaging methodology. Further, as noted above, the intracellular processes involved in apoptosis of RGC-5 cell culture (no astrocytes) under pressure have been studied by several groups [10], [14]-[16] and these provide guidance to our studies. During the course of our study, the authors become aware of the work of Van Bergen et al. [23] that cast uncertainty on whether the origin of the RGC-5 cell line is rat or mouse (although, elevated pressure is known to induce RGC apoptosis in both species [11], [24]). Thus, to ensure the validity of our results, only those results obtained using assays independent of the species origin of the cell (cell morphology, Ca^2+^ elevation, and caspases-3/7) are reported here.

The RGC-5 cells were cultured and differentiated using protocols described in the Supporting Information S1. The differentiated RGC-5 cells are used throughout this study because their physiological characteristics are even closer to primary RGCs compared to the undifferentiated ones [25].

**Figure 1:**
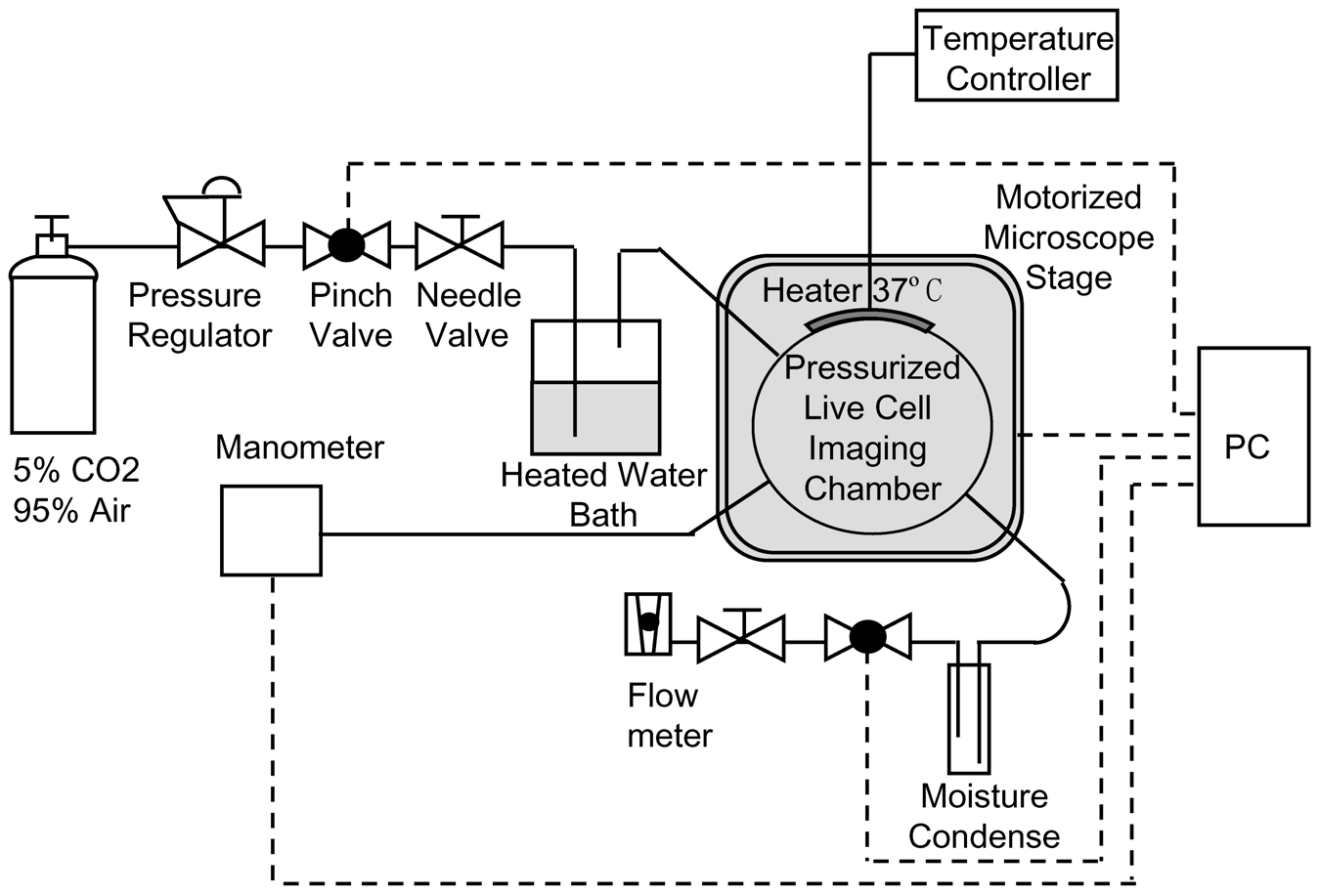
Schematic of imaging system. Schematic showing the custom-designed automated imaging system for real-time imaging of multiple cellular processes in individual live cells under elevated pressure.

For the real-time imaging study of RGC apoptosis under elevated hydrostatic pressure, a pressurized incubation chamber was custom-designed and built. The schematic of the imaging system is shown in Fig. 1. A photograph of the pressurized chamber on the stage of an Olympus IX71 inverted optical microscope is shown in Fig. S1A. A cross-sectional schematic showing the detailed design of the chamber is presented in Fig. S1B. To improve the throughput of real-time imaging, an automated microscopy system was established to allow access to a significantly larger area in a cell culture (beyond the field of view of an objective lens) in a single experimental run via time-multiplexing. The detailed design of the pressurized incubation chamber and the automated microscopy system is presented in the Supporting Information S1. In a typical experiment, such as designed to image intracellular Ca^2+^ elevation, caspase-3/7 activation, and morphological change during pressure-induced apoptosis in differentiated RGC-5 cells, ∼10 different locations in the cell culture are routinely imaged in transmission and fluorescent modes at programmable time intervals over times that are of relevance for the dynamics of observed molecular processes during the cell apoptosis. Reported results here were typically acquired at 5 minutes interval for over 20 hours, generating ∼10,000 images in a single run. Caspase-3/7 and Ca^2+^ activities were imaged using caspase-3/7 sensitive MR-(DEVD)_2_ and Ca^2+^-sensitive Fluo-4 AM fluorescent probes, respectively. The protocols for the probe loading and imaging are provided in the Supporting Information S1.

The results of cell apoptosis under elevated pressure (100 mmHg) reported in this paper were acquired in the following three sets of experiments. In the first set morphological changes and caspase-3/7 activation were measured in a population of 31 cells in a single run. In the second set morphological change and intracellular Ca^2+^ are imaged in a total of 23 cells in two runs. In the third set Ca^2+^, caspase-3/7, and morphological changes were imaged in three runs of roughly 19 cells each for a total of 56 cells. Correspondingly, four control experiments falling in three sets at 15 mmHg were conducted: one run of morphological changes and caspase-3/7 activation (20 cells), one run of morphological change and intracellular Ca^2+^ (14 cells), and two runs of Ca^2+^, caspase-3/7, and morphological changes (43 cells in two runs).

Additionally, we carried out experiments to identify and examine interference effects between the use of fluorophores for Ca^2+^ and caspase-3/7 and established imaging conditions for preventing phototoxic effects on cell death. Unlike imaging one biomarker at a time, the real-time simultaneous imaging of multiple processes using multiple fluorescent markers inevitably faces additional challenges of avoiding interference effects as each fluorescent marker is now exposed not only to the light excitation intended for it but also the excitation wavelengths necessary for other markers. Thus unanticipated adverse effects due to such cross-light excitation can occur and experimental checks need to be carried out to avoid such interference. During the investigations we found that excitation of MR-(DEVD)_2_ at 488 nm wavelength (the excitation wavelength for Fluo-4) can induce unintended cell death if the total dosage of the 488 nm light exceeds a threshold. Thus experiments for assessing the phototoxicity of the biomarkers during simultaneous Ca^2+^ and caspase-3/7 imaging were conducted. We measured the percentage of MR-(DEVD)_2_-loaded RGC-5 cells which undergo apoptosis at zero pressure under varied 488 nm excitation dosages and determined the dosage below which the normal healthy cell condition is not affected. The 488 nm excitation dosage in all simultaneous Ca^2+^ and caspase-3/7 imaging experiments described is thus kept safely below the measured threshold. Furthermore, to most effectively use the allowable 488 nm excitation dosage, we imaged Ca^2+^ at short (5 minutes) time intervals to capture the transient behavior of Ca^2+^ expected during the 0 to 8 hour time window as found in the experiments in which only Ca^2+^ was imaged at 5 min interval for 20 hours. After 8 hours the Ca^2+^ was imaged at 30 minute intervals.

## Results

### Morphology and Caspase-3/7 imaging

**Figure 2:**
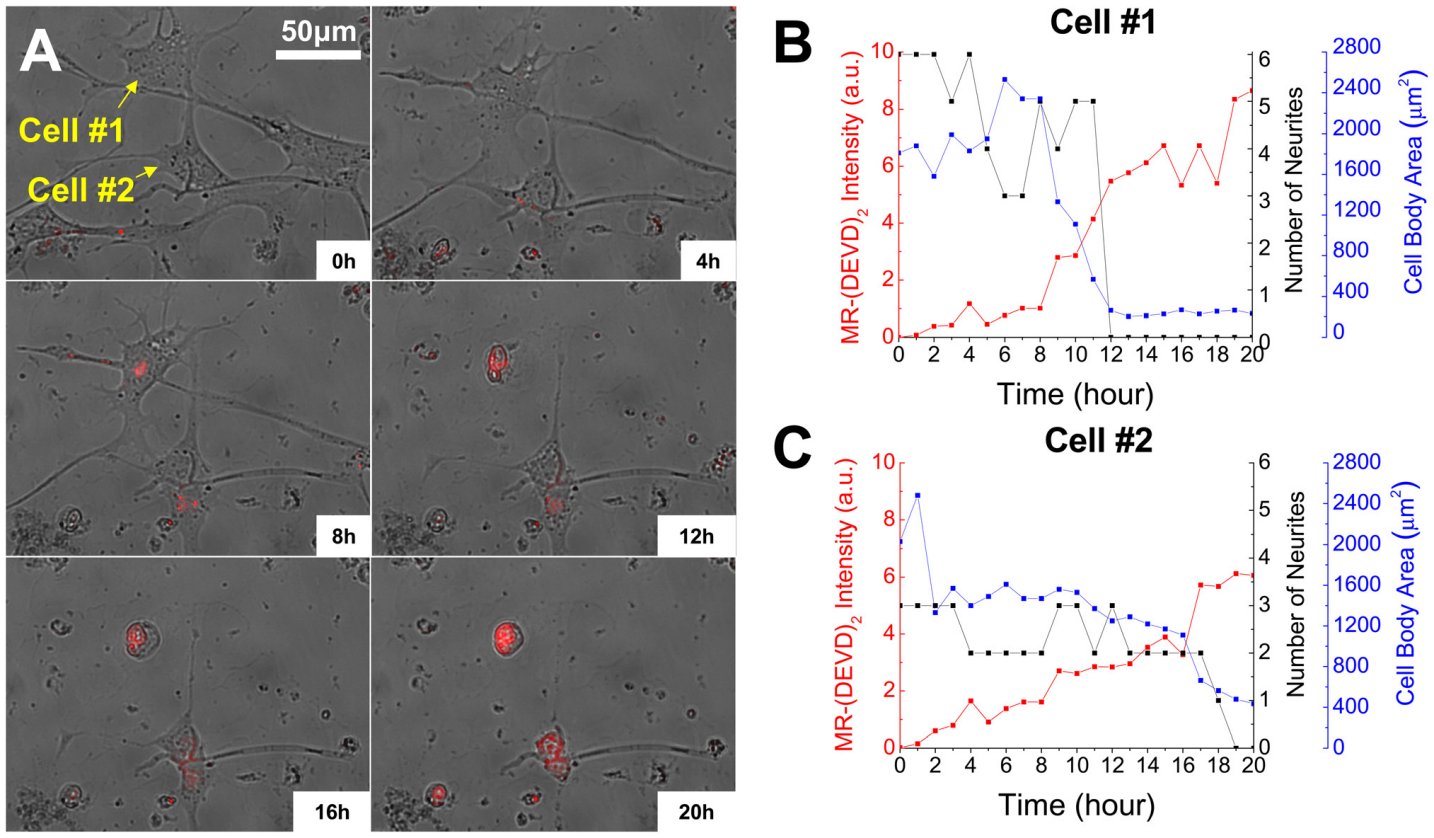
Simultaneous morphology and caspase-3/7 imaging. (A) Superimposed time-lapsed images showing the morphological change and caspase-3/7 activation in two individual differentiated RGC-5 cells (cell #1 and cell #2) going through apoptosis under 100 mmHg over 20 hours. The images are composed by overlapping phase-contrast image and red fluorescence image of cells loaded with MR-(DEVD)_2_. Plots for MR-(DEVD)_2_ fluorescence intensity, neurites number, and cell body area for cell #1 (B) and #2 (C) shown in (A).

We first present findings on simultaneous imaging of cell morphological changes (neurite retraction and cell body area reduction) and effector caspase-3/7 activation. A movie showing the changes in cell morphology and effector caspase-3/7 activation under 100 mmHg pressure (the intra-ocular pressure (IOP) in acute glaucoma) over twenty hours is provided in the Supporting Information (Movie S1). The corresponding control experiment of simultaneous imaging of morphology and caspase-3/7 was carried out at 15 mmHg (normal IOP). The morphology of the monitored 20 cells exhibited normal random increase/decrease in cell body area and neurite number. Only 3 out of 20 cells exhibited spontaneous morphological changes of cell body shrinkage and neurite retraction. No noticeable caspase-3/7 activation in the cell population was observed. Under elevated pressure of 100 mmHg, 20 cells out of the 31 cells showed apoptotic morphological changes (cell body shrinkage and neurite retraction). In Fig. 2A is shown an illustrative collection of super-imposed images of the cell morphology and the caspase-3/7 activation (red fluorescence) in two individual differentiated RGC-5 cells (labeled Cell #1 and #2 to facilitate discussion) taken at the same selected times over twenty hours under 100 mmHg. From such real-time imaging it is evident that, under elevated pressure, these two cells undergo characteristic apoptosis morphological changes, including neurites retraction and cell body area decrease as quantitatively plotted in Fig. 2B and C. The time of the characteristic morphological change of the number of neurites dropping to zero is ∼12 hours for cell #1 and ∼18 hours for cell #2, respectively, after the onset of pressure. Note that for both these cells the change in the MR-(DEVD)_2_ fluorescence intensity (corresponding to caspase-3/7 activation) up to ∼8 hours is similar but deviates measurably beyond, rapidly rising in cell #1 whereas remaining lower for cell #2. Indeed, considerable cell-to-cell variation in apoptotic morphological changes and caspase-3/7 activation is observed in the 31 cells imaged in this one run.

**Figure 3:**
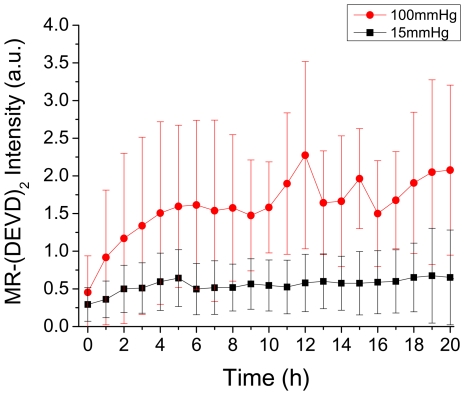
Population-averaged comparison of caspase-3/7 activation at 100 mmHg and 15 mmHg. Comparison of caspase-3/7 activation between cells under 100 mmHg (red circle, *n* = 31) and 15 mmHg (black square, *n* = 20). Results are shown as averaged fluorescence intensity of caspase-3/7 probe MR-(DEVD)_2_ ± standard deviation. The solid lines are only guide to the eyes.

In order to reveal how the real-time imaging of large numbers of individual cells complements the traditional paradigm of presenting the averaged behavior of lysed or fixed cells, in Fig. 3 (red circles) is shown the averaged MR-(DEVD)_2_ fluorescent intensity as a function of time for the 31 cells measured individually. In the corresponding control experiment at 15 mmHg in which only caspase-3/7 and cell morphology was imaged, the MR-(DEVD)_2_ fluorescence intensity reveals that at this pressure the caspase-3/7 level in the RGC-5 cells does not increase indicating the lack of apoptosis. It is evident that while a measurement of the average cell behavior is very useful to check the involvement of certain cellular processes (such as, in this case, caspase-3/7 activation), it fails to provide information in two important aspects: (1) the level of variation in the cell behavior as reflected by the size of the error bars in the plots of Fig. 3, and (2) what connection, in spite of such large variation, exists between individual cellular processes, such as between caspase activation and morphological change in the same cell, or between intracellular Ca^2+^ and morphological change as discussed below.

### Dynamics of intracellular Ca^2+^ during RGC apoptosis

**Figure 4:**
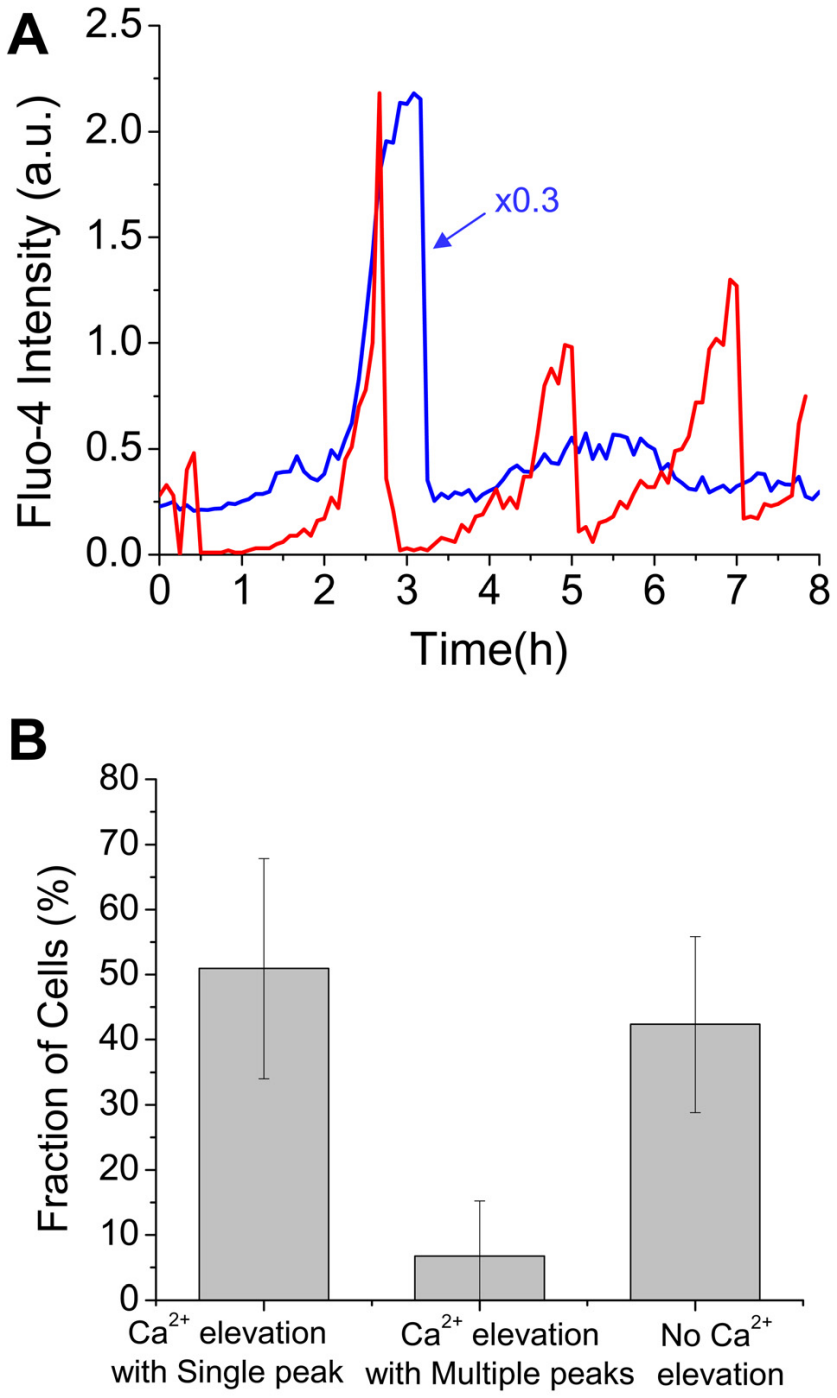
Dynamics of intracellular Ca^2+^ in RGC apoptosis. (A) Illustrative examples of intracellular Ca^2+^ dynamics in two individual differentiated RGC-5 cells during apoptosis under 100 mmHg pressure. One cell shows single Ca^2+^ peak (blue line) and the other shows multiple peaks (red line). Note that the Ca^2+^ elevation shows two-stage process of gradual increase over about one to two hours followed by a short transient peak of about 0.5 hour duration. (B) The fraction of cells showing Ca^2+^ elevation with a single peak, with multiple peaks, and no Ca^2+^ elevation. Values are mean ± standard deviation from 5 independent experiments.

From the generic important role that Ca^2+^ is known to play in regulating and amplifying apoptosis signaling through mitochondria and ER [17], it is expected that Ca^2+^ signaling is involved in pressure-induced RGC apoptosis. Some indirect hints were provided by Das et al. who found that cultured RGC-5 cells overloaded with Ca^2+^ due to treatment by ionomycin undergo apoptosis through intrinsic mitochondrial pathway [26] and by Cellerino et al. [27] who observed Ca^2+^ elevation in apoptotic RGCs in explanted retina. More direct evidences of the involvement of Ca^2+^ under elevated pressure were provided by Sappington et al. [28] who reported that Ca^2+^ elevation was observed in primary RGCs at about one hour after pressurized at 70 mmHg and by Niittykoski et al. [29] reporting that basal Ca^2+^ level increases in RGCs after two weeks under pressure of ∼30 mmHg. None of these studies provides however the real-time dynamics of Ca^2+^ in individual RGCs under elevated pressure. Below we show the first direct measurements of intracellular Ca^2+^ dynamics in RGC apoptosis under elevated pressure. Figure 4A shows the temporal behavior of intracellular Ca^2+^ in two individual differentiated RGC-5 cells under 100 mmHg. Transients in Ca^2+^ elevation with single (Fig. 4A (blue line)) or multiple peaks (Fig. 4A (red line)) are observed at an early stage of RGC apoptosis. A total of 46 cells (58%) out of 79 exhibited Ca^2+^ elevation. Among these 46 cells, 39 cells showed Ca^2+^ elevation with a single peak and 7 cells with more than one Ca^2+^ peak (Fig. 4B). In the control experiment (15 mmHg), none of the cells exhibited Ca^2+^ signal characteristic of apoptosis. The dynamics of intracellular Ca^2+^ elevation during 100 mmHg pressure-induced apoptosis is observed to be characterized by two features: a gradual increase in Ca^2+^ over approximately one to two hours, followed by a transient with a typical full width at half maximum of ∼20 minute duration.

### Simultaneous imaging of Ca^2+^, caspase-3/7, and the morphological change

Next we present results of experiments in which the molecular processes of intracellular Ca^2+^ elevation and caspase-3/7 activation, as well as the morphological changes (neurites number and cell body area), were monitored simultaneously under 100 mmHg. Indeed, although Ca^2+^ is known as a universal regulator/initiator/amplifier of apoptosis and caspase-3/7 as a final effector of apoptosis [17], [18] in general, the difference in the dynamics of the two processes has not been addressed previously.

**Figure 5:**
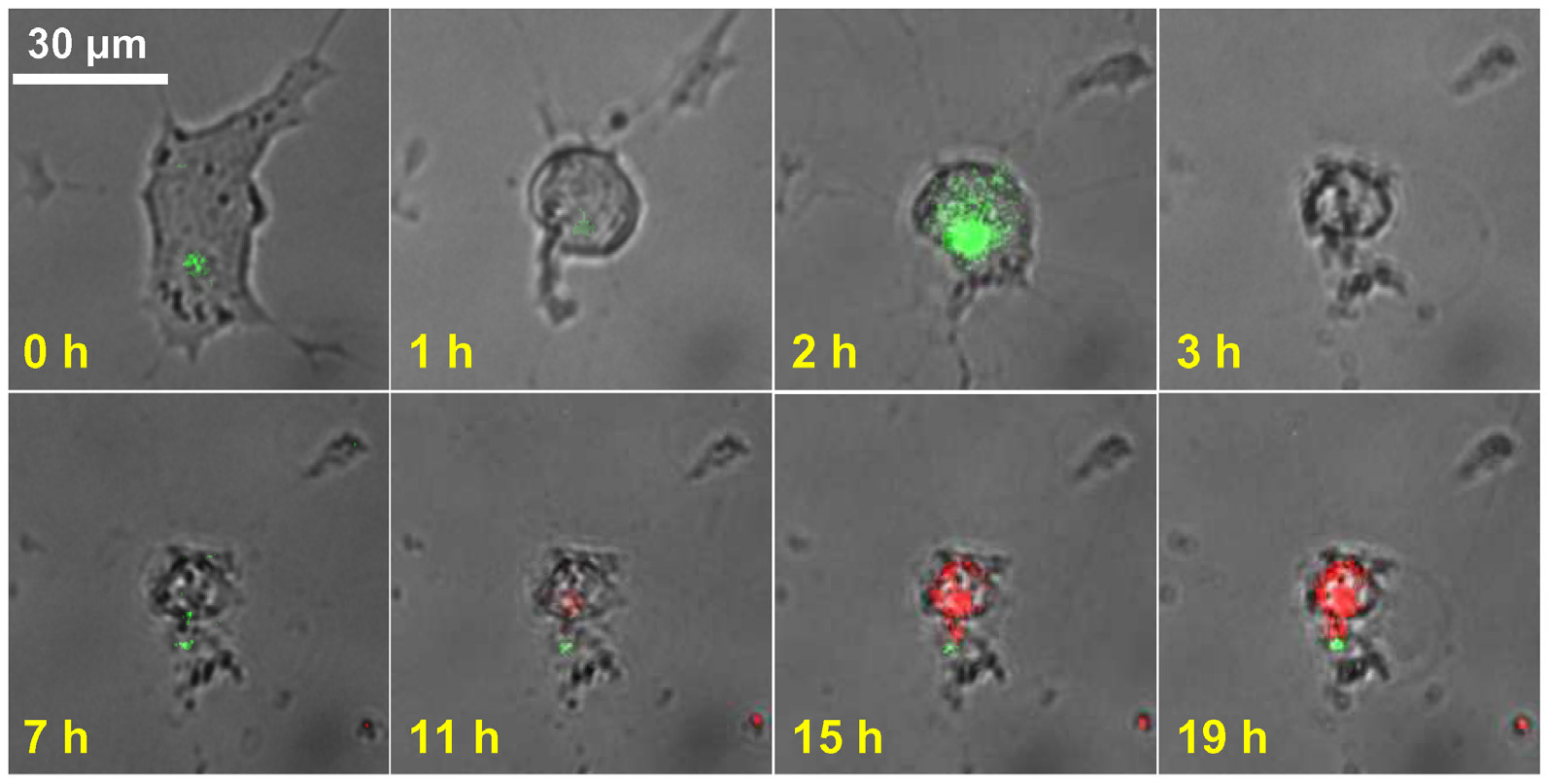
Simultaneous imaging of Ca^2+^, caspase-3/7, and morphological change. Superimposed time-lapsed images illustrating the morphological change (grey phase contrast), Ca^2+^ elevation (green fluorescence), and caspase-3/7 activation (red fluorescence) in a differentiated RGC-5 cell going through apoptosis under 100 mmHg taken at the marked hours after the onset of pressure elevation.

**Figure 6:**
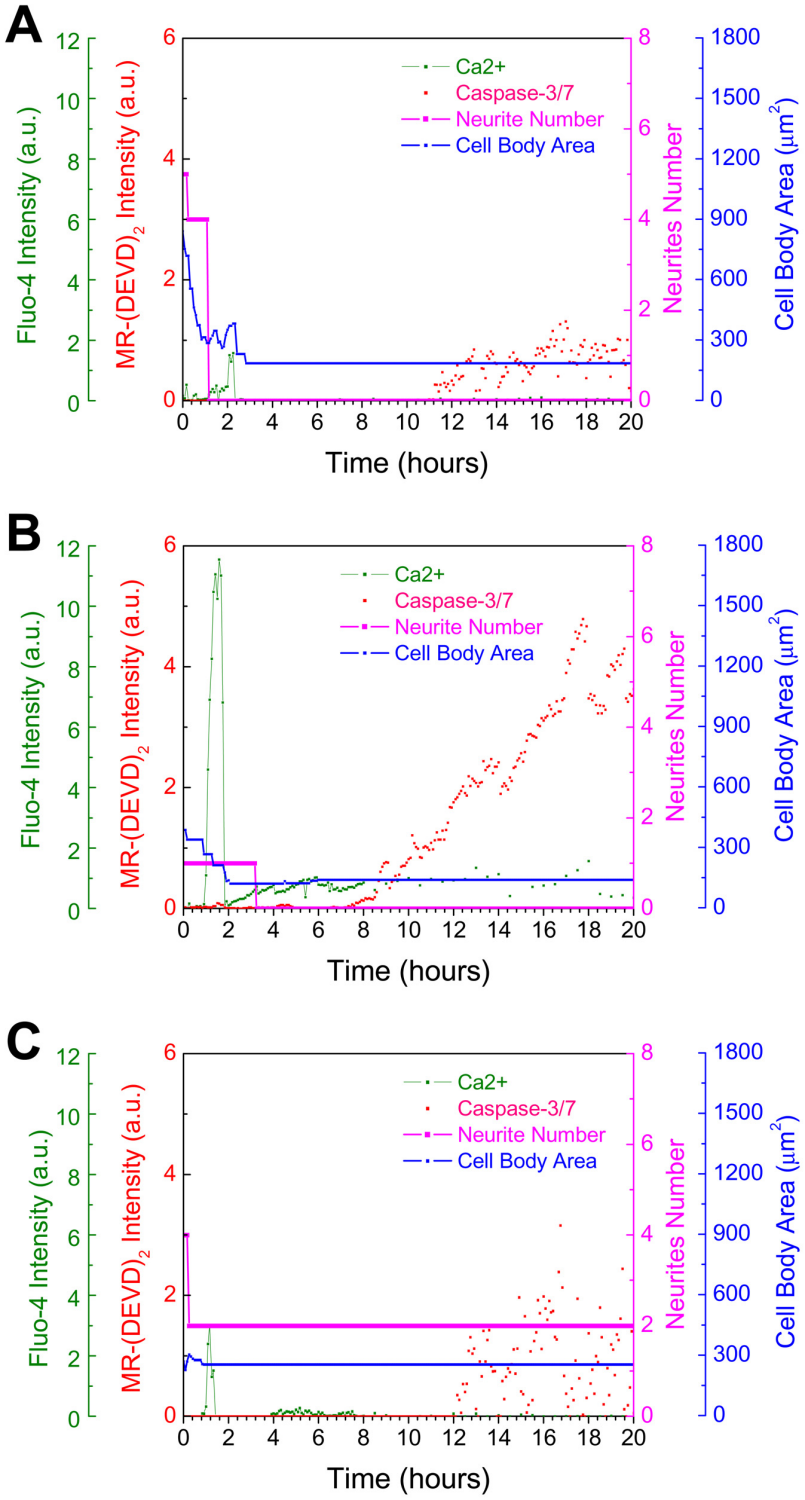
Measured Ca^2+^ elevation, caspase-3/7 activation and morphological changes in individual cells. Illustrative plots of Ca^2+^, caspase-3/7, cell body area, and neurite number in three different cells under 100 mmHg. Panel (A) shows the associated plots of the time-lapse images of the differentiated RGC-5 cell under 100 mmHg inFig. 5 showing Ca^2+^ peak at the early stage around 2 hour followed by caspase-3/7 activation at the late stage around 11 hour. The plots of the cells in (B) and (C) illustrate the cell-to-cell variation in the time interval between Ca^2+^ elevation and onset of caspase-3/7 activation. Although Ca^2+^ peaks occur around 2 h in the both cells, caspase-3/7 is activated around 8 h in the cell in (B) and around 12 h in the cell in (C).

Figure 5 shows an illustrative set of superimposed time-lapsed fluorescence images of Ca^2+^ (green), caspase-3/7 (red), and phase contrast images (grey) of the morphological changes of neurites number and cell body area in a differentiated RGC-5 cell under elevated pressure of 100 mmHg. The corresponding plots of the Ca^2+^, caspase-3/7, neurites number, and cell body are shown in Fig. 6A. The transient peak of Ca^2+^ with a duration of ∼15 minutes was observed at an early stage of around 2 hours, followed by a gradual activation of caspase-3/7 at a later stage of around 11 hours in this cell. The cell also shows the typical apoptotic morphological changes including cell body area reduction and neurite number decrease at the relatively early time of around 1 to 2 hours after the onset of the 100 mmHg pressure. We note that in the two runs of control experiment of Ca^2+^, caspase-3/7, and morphology imaging under 15 mmHg (normal IOP) none of the monitored 43 cells showed noticeable Ca^2+^ elevation or caspase-3/7 activation over the same period of 20 hours. Only 7 out of the 43 cells exhibited spontaneous morphological changes of cell body shrinkage and neurite retraction. Under apoptosis, a significant cell-to-cell variation in the time interval between Ca^2+^ elevation and caspase-3/7 activation was observed. Figure 6B and C are illustrative of the cell-to-cell variation under identical environment of 100 mmHg pressure. The cell in Fig. 6B exhibits a Ca^2+^ peak at around 2 hours and caspase-3/7 activation at ∼8 hours after pressure elevation whereas the cell in Fig. 6C also exhibited the Ca^2+^ peak at around 2 hours but the caspase-3/7 activation occurred at ∼12 hours.

**Figure 7:**
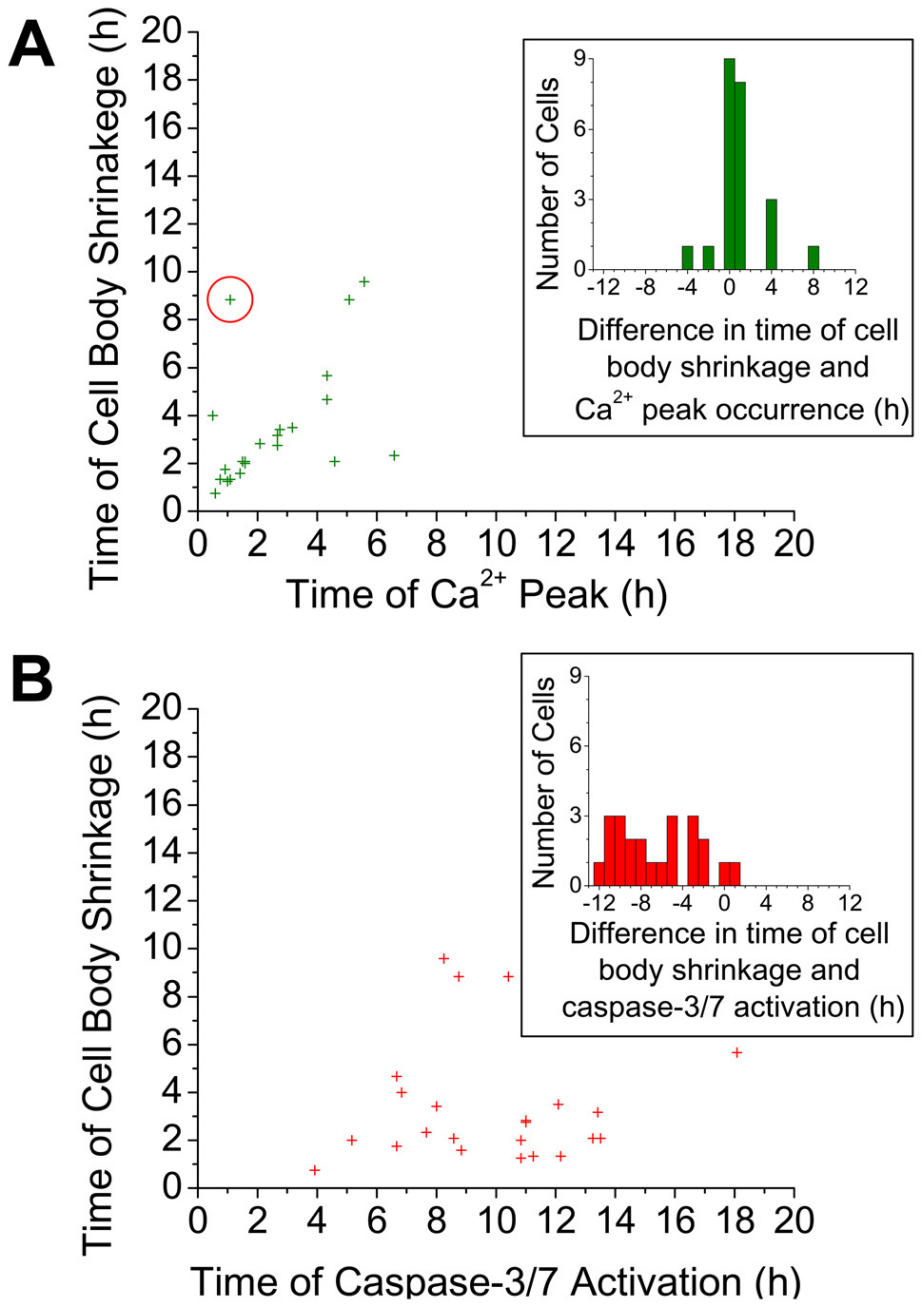
Comparison of the time of cell morphological changes vs. Ca^2+^ peak occurrence and caspase-3/7 activation. Plots of the time of cell body shrinkage versus (A) time of Ca^2+^ peak occurrence and (B) time of caspase-3/7 activation. Insets show the distribution of time difference between the time of cell body shrinkage and (A) the Ca^2+^ peak occurrence and (B) caspase-3/7 activation. Note that in (A) two data points at coordinate (1.58, 2) overlap. Red circles indicate outliers identified using generalized ESD outlier test (see text). The correlation coefficients between the time of cell body shrinkage versus the time of Ca^2+^ peak occurrence is 0.66 (significance *p* = 1E-3) and versus the time of caspase-3/7 activation is 0.06 (*p* = 0.78).

Under 100 mmHg, from three experimental runs, a total of 56 cells were monitored for 20 hours. Amongst the 56 cells imaged, 21 cells exhibited neither Ca^2+^ elevation nor caspase-3/7 activation. Thirty two cells exhibited Ca^2+^ elevation with apoptotic cell morphological changes of cell body shrinkage and/or neurites retraction. All these 32 cells subsequently exhibited caspase-3/7 activation. In addition, 3 cells exhibited caspase activation without detectable Ca^2+^ elevation. Among the 32 cells, 23 cells exhibited both cell body shrinkage and retraction of all neurites. The time of the occurrence of cell morphological change (cell body shrinkage and neurite retraction) versus the two chemical processes (Ca^2+^ elevation and caspase-3/7 activation) in the 23 cells is summarized in Fig. 7 and Fig. S2. Fig. 7 shows the scatter plots of the time of cell body shrinkage versus the time of occurrence of the Ca^2+^ peak (Fig. 7A) and versus the time of the onset of significant caspase-3/7 activation (Fig. 7B). The insets show the cell distribution as a function of the time difference between cell body shrinkage and Ca^2+^ peak occurrence (Fig. 7A) and caspase-3/7 activation (Fig. 7B). In the few cells where more than one Ca^2+^ peak is observed, we took the first peak which in all cells we found to be the dominant as well. The time of cell body shrinkage is defined as the time at which the cell body area has reached within ∼10% of the final area. Correspondingly, in the Supporting Information we show (Fig. S2) the scatter plots of the time of neurite retraction (defined by the time in which the neurite number becomes zero) versus the time of the Ca^2+^ peak (Fig. S2A) and versus the time of caspase-3/7 activation (Fig. S2B), both from the same 23 cells. The insets in Fig. S2 show the behavior as a function of the time difference between the processes. The time difference between Ca^2+^ peak and cell body shrinkage (Fig. 7A inset), and between Ca^2+^ peak and neurite retraction (Fig. S2A inset) is distributed at 0±1 hour in 74% (cell body shrinkage) and 78% (neurite retraction) of the cells. This is in clear contrast to the difference of the time of cell body shrinkage or neurite retraction and caspase-3/7 activation (Fig. 7B and Fig. S2B insets) which is widely distributed among the 23 cell.

Analysis of the data of Fig. 7 and Fig. S2 is provided in Supporting Information S1 with the key results summarized in Table S1 in Supporting Information S1. Here we note that there are apparent outlier data points in Fig. 7A and Fig. S2A, potentially manifestations of the spontaneous cell body shrinkage and neurite retraction found in the 15 mmHg control experiments (i.e. unrelated to pressure-induced apoptosis) as noted above. Thus a generalized extreme studentized deviate (ESD) test [30], [31] was employed to identify such outliers. One outlier in Fig. 7A and three outliers in Fig. S2A were so identified with 95% confidence level. No outliers could be identified in Fig. 7B and Fig. S2B. The time of cell body shrinkage and neurite retraction is correlated positively and well with the time of Ca^2+^ peak occurrence (correlation coefficient *r* = 0.66 and significance *p* = 1E-3 between time of cell body shrinkage and Ca^2+^, and *r* = 0.85 and *p* = 2E−6 between time of neurite retraction and Ca^2+^). However the time of cell body shrinkage and neurite retraction is poorly correlated with the time of caspase-3/7 activation (*r* = 0.06 and *p* = 0.78, and *r* = 0.29 and *p* = 0.18, respectively). Moreover, Student's t-test establishes with 99.99% confidence level that the morphological changes of cell body area and neurite retraction precede the observation of significant activation of caspase-3/7 (see Supporting Information S1). The results above are consistent with the suggestion in Ref. [21] that ion channel activities are responsible for the apoptotic morphological changes without significant caspase-3 activation (See Discussion section for detail).

**Figure 8:**
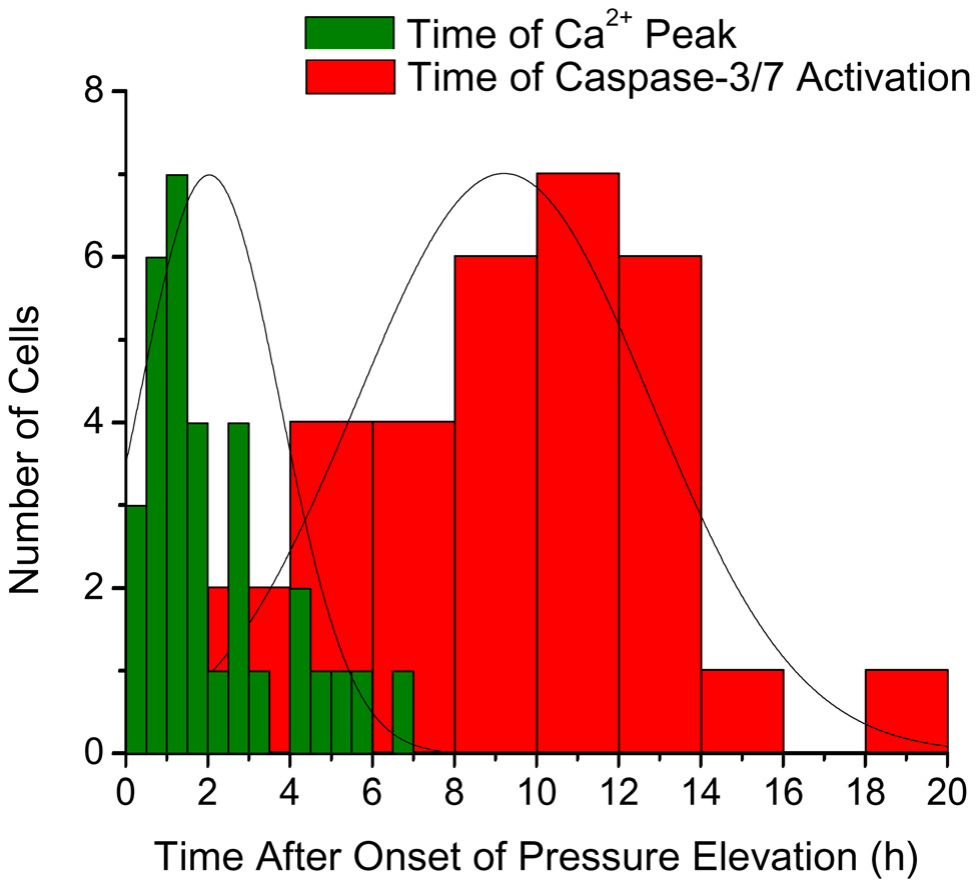
Distribution of the time of Ca^2+^ peak occurrence and caspase-3/7 activation in the cell population. A histogram showing the distribution of the times of Ca^2+^ peak and caspase-3/7 activation in 32 differentiated RGC-5 cells undergoing apoptosis under elevated pressure.

Furthermore, we find that the Ca^2+^ elevation always precedes significant rise in caspase-3/7 concentration although the interval between them varies from ∼1 to ∼14 hours. In Fig. 8 we summarize the observed dynamics of Ca^2+^ elevation and caspase-3/7 activation in all 32 cells in which both processes are observed. The mean time and standard deviation of Ca^2+^ peak occurrence are 2 hours and 1.7 hours, respectively. By contrast, the mean time and standard deviation of the onset of caspase-3/7 activation are 9.2 hours and 3.6 hours, respectively. Thus Ca^2+^ elevation is narrowly distributed at the early stage of apoptosis while caspase-3/7 activation is relatively widely distributed around a late time in the apoptosis process. Subject only to the quantitatively unknown threshold concentration of caspase-3, our findings seem to suggest that caspase-3 presence is not required for Ca^2+^ elevation, consistent with the conclusion of Ref. [3] but unlike the suggestion in Ref. [19]. The cell-to-cell variation in the interval between these molecular processes during apoptosis is further discussed below, as is the temporal correlation between the morphological changes and responsible intracellular molecular process.

## Discussion

### Origins of Ca^2+^ dynamics during RGC apoptosis

Intracellular Ca^2+^ plays an universal role as regulator of several cell functions such as motility, growth cell cycle, endo/exocytosis, death, glucose metabolism, etc. [32]. Recently, the role of Ca^2+^ in apoptosis has attracted attention as a regulator/amplifier of apoptosis signaling [33]. For understanding the time-dependent nature of apoptosis signaling pathways, it is thus important to extract information on the temporal behavior of Ca^2+^ dynamics in real-time in living cells undergoing apoptosis. It is well established that in normal cells the dynamics of intracellular Ca^2+^ is governed by a balance of fluxes between the intracellular stores of Ca^2+^, i.e. the ER and the mitochondria, and the flux between the cytosol and the extracellular medium [34]. The oscillating behaviors of the Ca^2+^ dynamics as a result of the balanced Ca^2+^ fluxes in normal cells under physiological condition have been examined experimentally as well as through mathematical modeling [35], [36]. In our study of RGC apoptosis due to elevated pressure, two distinctive characteristics of Ca^2+^ dynamics are observed: (1) a temporal profile of a gradual elevation over about one to two hours followed by a rapid increase and a peak of ∼20 minutes duration; and (2) although the majority (∼85%) of apoptotic cells expressed a single Ca^2+^ peak, some exhibited multiple peaks of Ca^2+^.

Different temporal profiles of Ca^2+^ elevation in apoptosis, including sustained increase, single transient peak, and multiple peaks, have been reported in different apoptotic cells under different apoptosis inducers [37]–[39]. The origin of the different Ca^2+^ temporal profiles during apoptosis has, however, remained unresolved. Some insight into the origin of single versus multiple peaks during apoptosis can be obtained from the studies by Li et al. [40] of Ca^2+^ oscillation under normal physiological condition. These authors employed mathematical modeling to examine the observed Ca^2+^ behavior in normal cells and concluded that the origin of the single or multiple peaks resides in the different IP3 concentrations. It was found that the frequency of the Ca^2+^ oscillation increases as the concentration of cytosol IP3, an agonist of IP3 channel, increases. However, at extremely high stimulus of IP3, Ca^2+^ shows a broad single peak rather than oscillatory behavior. In light of this, a potential origin of our observation of the mixture of single and multiple peaks may lie in different IP3 concentration dynamics as the various apoptotic processes progress under stress. Our findings suggest that measurement of the IP3 concentration in apoptotic cells would shed valuable light on the issue of the role of IP3 concentration level in impacting the occurrence of single versus multiple Ca^2+^ peaks.

Next we turn to the observed gradual elevation of Ca^2+^ over about one to two hours before the rapid increase and occurrence of a peak (Fig. 4). Although, as noted above, a complete model for elucidating the full Ca^2+^ dynamics in apoptotic cell has not been established yet, the literature does provide valuable insight into the intracellular processes that are contributing to the dynamics of Ca^2+^ under apoptosis. A pioneering investigation of the mechanism of regulating Ca^2+^ elevation in apoptosis was carried out by Boehning et al. that examined the dynamics of Ca^2+^ and another key player in apoptosis: the cytochrome c released from the mitochondria [3]. These authors showed that Ca^2+^-induced inhibition of Ca^2+^ release from the ER through the IP3 receptor/channel (negative feedback for preventing overloading of Ca^2+^ in cytosol) is blocked by the binding of the mitochondria released cytochrome c to the IP3 receptor/channel, thus leading to a massive Ca^2+^ release from the ER into the cytosol through the IP3 receptor/channel. Our observed rapid rise of Ca^2+^ may thus originate from this cytochrome c-promoted Ca^2+^ flux from the ER during the early stage of apoptosis. Further investigations are needed for the clarification of the origin of the behavior of Ca^2+^ in apoptosis, including spatially-resolved measurements of the dynamics of Ca^2+^ in the ER, the cytosol, and the mitochondria, as well as the release of cytochrome c from the mitochondria, coupled with mathematical modeling based upon the measured data.

### Concurrence of Ca^2+^ elevation with apoptotic morphological changes

Two views are found in the literature concerning the molecular level process responsible for the apoptotic morphological change of cell body shrinkage. The more common view is that caspase-3 causes the apoptotic morphological changes through the cleavage of cytoskeleton proteins or cytoskeleton protein-associated proteins supporting the structure of the cell [20]. The other view is that efflux (from the cytosol to the extracellular medium) of ions including K^+^, Cl^−^, Na^+^, etc. through ion channels in the plasma membrane induces cell body shrinkage as it causes abnormal osmotic pressure leading to water efflux [21]. Under apoptosis, the intracellular signaling molecule(s) inducing such ion efflux, and thus leading to the cell body shrinkage, has not been clarified yet. However, Ca^2+^ has been shown to play a role in regulating these ions channels in apoptotic cells. Nietsch et al. showed that the decrease of Ca^2+^ with Ca^2+^ chelator EGTA (ethylene glycol tetraacetic acid) inhibits the K^+^ and Cl^−^ channel currents in tumor necrosis factor (TNF)-induced apoptosis in HTC rat hepatoma cells, suggesting that these ion channel activities during apoptosis are Ca^2+^-dependant [41]. Krick et al. demonstrated that the ionophore FCCP (Carbonyl cyanide-p-trifluoromethoxyphenylhydrazone) induces the cytoplasmic Ca^2+^ increase, leading to the opening of Ca^2+^-activated K^+^ channels in apoptotic pulmonary artery smooth muscle cells [42]. These papers suggest that intracellular Ca^2+^ would be a regulator of ion channel activities during apoptosis.

Our studies of real-time dynamics of apoptosis provide, for the first time, data that shed valuable light on the above noted two views concerning molecular processes underlying apoptotic morphological changes. As noted in the Results section, the time of the cell morphological changes (cell body area and neurite retraction) is correlated with and essentially coincides with the time of Ca^2+^ elevation. In contrast, the time of morphological change is poorly correlated with and significantly precedes the time of capase-3/7 activation. These findings indicate that caspase-3/7 is not likely to cause the early morphological changes. Thus our simultaneous imaging data appear to provide support for the view in the literature [21] that the ion channel activity leading to water efflux from the cell is responsible for the early apoptotic morphological changes in RGC-5 under elevated hydrostatic pressure without the need for significant caspase-3/7 activation (i.e. unless the background level of caspase-3 is sufficient to play a significant role for which, at this point, no sufficiently discriminatory testable hypothesis can be offered).

### Time distribution of Ca^2+^ elevation and caspase-3/7 activation: The apoptotic pathway

Regarding the connection between the time of Ca^2+^ elevation and caspase-3/7 activation, as summarized in Fig. 8, the former is narrowly distributed at the early stage of apoptosis (2±1.7 h) while the latter is relatively widely distributed around a late time in the apoptosis process (9.2±3.6 h). There is also a significant cell-to-cell variation in the time interval between Ca^2+^ elevation and caspase-3/7 activation as seen in Fig. 6B and C. Recall that pressure-induced RGC apoptosis is considered to be governed by the mitochondria-mediated apoptosis signaling pathway [15], [16]. As noted before, the complex mitochondria-mediated pathway involves the release of cytochrome c from mitochondrion into the cytosol, followed by apoptosome formation comprising the released cytochrome c and Apaf-1 in the cytosol with the assistance of dATP. The apoptosomes activate caspase-9, which in turn activates caspase-3/7, the effector caspase that eventually leads to the dismantling of the cytoskeleton. As discussed earlier in Sec IV.1, cytochrome c released from the mitochondria also affects the IP3 receptors on the ER and leads to the massive Ca^2+^ release from ER into the cytosol [3]. The delay we observe between the average times of Ca^2+^ peak and onset of caspase-3/7 activation (Fig. 8), as well as the variation in the time interval between Ca^2+^ peak and onset of caspase-3/7 activation are thus manifestations of the expected cumulative effect of fluctuations in the times of the many biochemical reactions involved between the cytochrome c release, apoptosome formation, and the caspase-3/7 activation. This is borne out by the kinetic Monte-Carlo simulation studies of Raychaudhuri et al. [43] that reveal large cell-to-cell variation in the caspase-3/7 activation for the mitochondria-mediated apoptosis as arising from the large fluctuations in the rate of apoptosome formation involving the kinetics of the above noted stochastic processes.

### Summary and Future Studies

Examining the dynamics of multiple molecular level processes in a large number of individual cells from the same live cell population under controlled stress allows the construction of statistically meaningful real-time temporal and spatial map of molecular processes necessary to gain deeper understanding of complex signal networks of cellular processes. The custom-designed imaging platform developed in our laboratory has enabled the start of the collection of such data for a large number of RGC-5 cells from a population undergoing apoptosis under controlled elevated hydrostatic pressure over prolonged times. This has already resulted in findings of considerable significance to the understanding of the intrinsic pathways of apoptosis. The simultaneous time-dependent behavior of intracellular Ca^2+^ elevation, caspase-3/7 activation, and morphological changes (cell area and number of neurites) in individual differentiated RGC-5 cells during apoptosis over twenty hours at 100 mmHg pressure are reported. The Ca^2+^ dynamics reveals a gradual initial increase followed by rapid peak of a short duration, typically less than 0.5 h. Moreover, our data reveal a strong temporal correlation between the appearance of the Ca^2+^ peak and morphological changes of neurite retraction and cell body area reduction at an early stage. The Ca^2+^ peak and morphological changes precede significant caspase-3/7 activation at later stage of apoptosis. These findings thus indicate that, as suggested by Maeno et al. [21], Ca^2+^ activity, through its impact on ion channel activity and water efflux, is likely responsible for the above noted early apoptotic morphological change during pressure-induced apoptosis. Furthermore, a significant cell-to-cell variation is found in the time interval between the intracellular Ca^2+^ elevation and the onset of caspase-3/7 activation. Such variation, also found in the kinetic Monte-Carlo simulations [43], reflects the stochastic nature of the intermediate biochemical processes connecting the intracellular Ca^2+^ elevation and caspase-3/7 activation during mitochondria-mediated apoptosis. However, the role of the low level of caspase-3/7 observed at the early stages when Ca^2+^ elevation and the above noted early morphological changes (neurite retraction and cell body area reduction) occur remains unclear. Our continuing efforts are aimed at establishing a database quantifying the distribution of the dynamics of a key set of apoptosis-related processes in a cell population under controlled stress that would serve as a foundation for the quantitative stochastic modeling of cell apoptosis.

In closing we note that while this particular work examines RGC apoptosis under elevated pressure, the development of the real-time imaging methodology to examine multiple cellular processes in a statistically significant set of individual cells from a cell population is motivated by the generic need to investigate multiple cellular processes in large numbers of live cells over prolonged periods of time under a broad spectrum of controlled stresses. We emphasize that, as a consequence of the stochastic nature of the underlying biochemical reactions, the progression of intracellular processes in individual cells under stress will exhibit an inevitable cell-to-cell variation which is not captured by conventional biochemical assays which measure population-averaged cellular responses. The dynamics of apoptosis from real-time live cell imaging reported here allows the true statistical nature of progression of different processes and, potentially critical, the interplay between them, to be directly revealed.

## Supporting Information

Figure S1 (A) Photograph of the pressurized cell incubation chamber on the motorized sample stage of an Olympus IX71 inverted optical microscope. (B) Cross-sectional schematic showing the detailed design of the chamber.
(4.87 MB TIF)

Figure S2 Plots of the time of neurite retraction versus (A) time of Ca^2+^ peak occurrence and (B) time of caspase-3/7 activation. Insets show the distribution of time difference between the time of neurite retraction and (A) the Ca^2+^ peak and (B) the caspase-3/7 activation. Red circles indicate outliers identified using generalized ESD outlier test. The correlation coefficients between the time of neurite retraction versus the time of Ca^2+^ peak occurrence is 0.85 (significance *p* = 2E−6) and that versus the time of caspase-3/7 activation is 0.29 (*p* = 0.18).
(1.60 MB TIF)

Movie S1 Real-time live cell simultaneous imaging of caspase-3/7 activation (represented by red fluorescence) and morphological change in differentiated RGC-5 cells under elevated pressure of 100 mmHg for 20 hours. One second play time in the movie corresponds to one hour in real time.
(1.36 MB AVI)

Supporting Information S1 Supplementary Materials and Methods, and Statistical Analysis.
(0.06 MB DOC)
