# Supplementary figures and images for: Real-Time Dynamics of Ca2+, Caspase-3/7, and Morphological Changes in Retinal Ganglion Cell Apoptosis under Elevated Pressure
Source: PLoS One. 2010 Oct 18;5(10):e13437. doi: 10.1371/journal.pone.0013437 (PMC2956638; doi:10.1371/journal.pone.0013437)

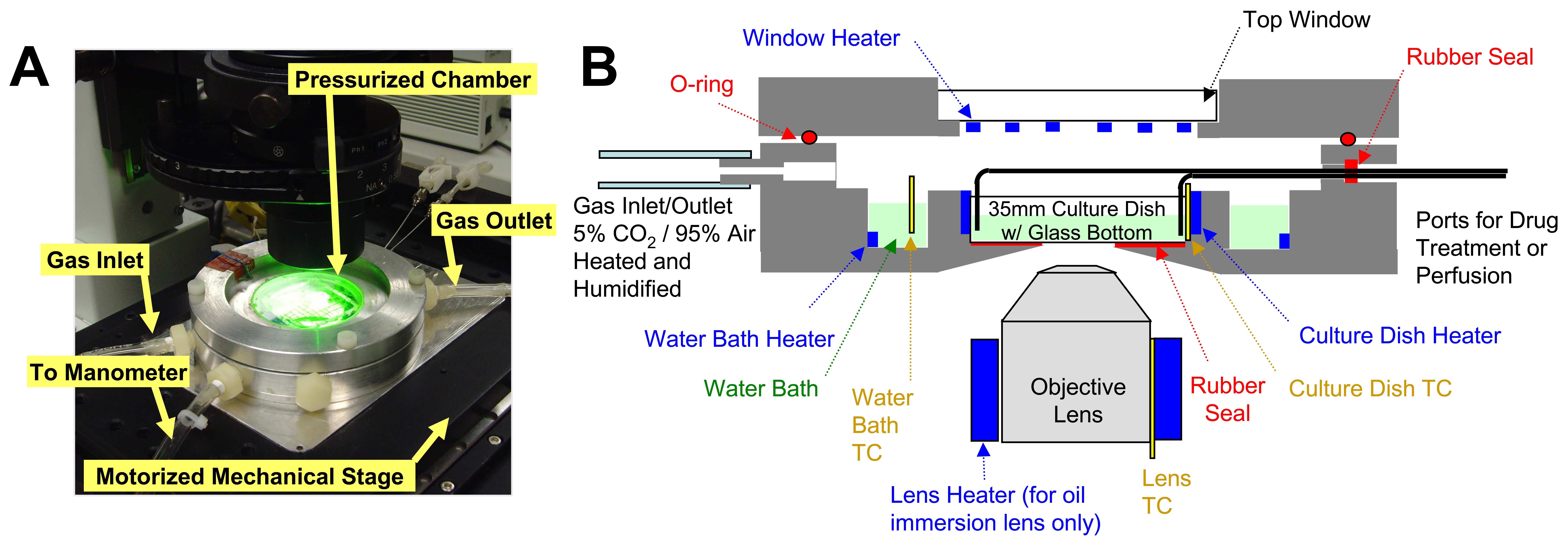

Supplement: Figure S1 — (A) Photograph of the pressurized cell incubation chamber on the motorized sample stage of an Olympus IX71 inverted optical microscope. (B) Cross-sectional schematic showing the detailed design of the chamber. (4.87 MB TIF) [file pone.0013437.s001.tif]

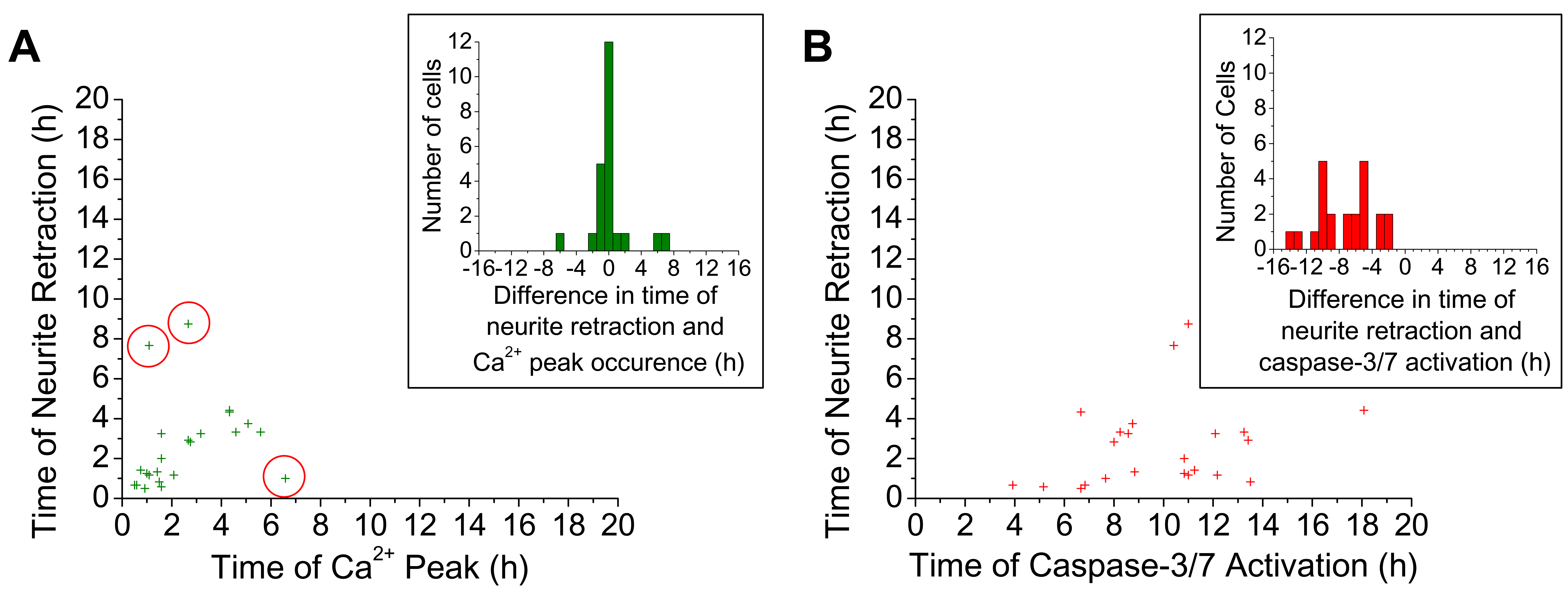

Supplement: Figure S2 — Plots of the time of neurite retraction versus (A) time of Ca2+ peak occurrence and (B) time of caspase-3/7 activation. Insets show the distribution of time difference between the time of neurite retraction and (A) the Ca2+ peak and (B) the caspase-3/7 activation. Red circles indicate outliers identified using generalized ESD outlier test. The correlation coefficients between the time of neurite retraction versus the time of Ca2+ peak occurrence is 0.85 (significance p = 2E−6) and that versus the time of caspase-3/7 activation is 0.29 (p = 0.18). (1.60 MB TIF) [file pone.0013437.s002.tif]
