# Supplementary material for: Real-Time Dynamics of Ca2+, Caspase-3/7, and Morphological Changes in Retinal Ganglion Cell Apoptosis under Elevated Pressure
Source: PLoS One. 2010 Oct 18;5(10):e13437. doi: 10.1371/journal.pone.0013437 (PMC2956638; doi:10.1371/journal.pone.0013437)
Supplement: Supporting Information S1 — Supplementary Materials and Methods, and Statistical Analysis. (0.06 MB DOC) [file pone.0013437.s004.doc]

**PLoS ONE**

**Supporting Information S1 for**

## Real-time Dynamics of Ca^2+^, Caspase-3/7, and Morphological Changes in Retinal Ganglion Cell Apoptosis under Elevated Pressure

Jae Kyoo Lee^1☯^, Siyuan Lu^2,3☯^, and Anupam Madhukar^1,2,4^*

**1** Departments of Biomedical Engineering, **2** Physics, **3** Ophthalmology, and **4** Materials Science

University of Southern California, Los Angles, CA 90089-0241, USA

 ☯These authors contributed equally to this work.

## Supplementary Materials and Methods

**RGC-5 cell culture and differentiation**

The RGC-5 cells were maintained in a growth medium containing DMEM (Gibco, 11885-076), 10% Fetal Bovine Serum, 44 mM NaHCO_3_, Penicillin (100 microgram/ml), Streptomycin (100 units/ml) and L-Glutamine (292 μgram/ml). The cells were subcultured every 3 days before use for the experiments. 35 mm culture dishes with Poly-L-Lysine coated glass coverslips as the bottoms were used for cell culture. Cells were seeded in the density of ~150 cells per mm^2^. The cells were grown for 24 hours before being differentiated using staurosporine (380-014, Alexis Biochemicals, San Diego, CA) dissolved in Dimethyl Sulfoxide (DMSO, Sigma, St. Louis, MO).

**Pressurized imaging chamber**

The pressure in the chamber was applied via a mixture of 95% Air + 5% CO_2_ gas as normally used for RGC-5 culture [S1]. The gas was pre-humidified by bubbling through a heated bottle of water. The chamber pressure was coarsely controlled by adjusting needle valves installed one each on the gas inlet and outlet side of the chamber. Finer pressure control is achieved via programmed opening and closing of pinch valves on the gas inlet and outlet according to the pressure value measured with a NIST traceable manometer (0.3 mmHg uncertainty) connected to a PC. For temperature control, four resistive heaters with corresponding thermocouples are used to heat, respectively, the cell culture dish, a water bath inside the chamber (to provide further control on humidity), the top window of the chamber, and the objective lens (if necessary). Additionally, in the pressure chamber, fluidic inlet and outlet are provided for the culture dish to allow perfusion of the culture media when the cells are incubated under pressure. This feature is particularly important for the study of the effect of neuroprotective drugs.

**Automated microscopy setup for real-time live cell imaging**

The pressure chamber, as noted above, is installed on the stage of an Olympus IX 71 inverted optical microscope for real-time imaging of the cells. Phase-contrast and fluorescent images are acquired using Olympus DP30BW Peltier-cooled CCD camera. The microscope is upgraded with a motorized xyz sample stage (MS-2000, ASI, Eugene, OR) on which the pressurized incubation chamber resides, a motorized filter turret, and motorized lamp shutters. The translation of the motorized stage in predefined cycles brings different locations of the cell culture into the field of view of the lens and allows these locations to be monitored in a time multiplexed cyclic fashion. All motorized components and the camera are controlled by a computer through a custom-designed software to allow programmable sequential imaging of multiple locations and multiple fluorescent-labeled biomarkers.

**Imaging of cell morphological change, caspase-3/7 activation, and Ca^2+^ elevation.**

The morphological changes in differentiated RGC-5 cells were studied by measuring the number of neurites per cell and cell soma size as a function of time. A protrusion from cell soma is counted as a neurite if its length is equal to or greater than the cell soma diameter as conventionally defined in the literature [S2,S3]. Only protrusions arising directly from the cell soma were counted as neurites. The measurement of the cell soma area was performed by manually drawing the boundary of the each cell soma on the phase-contrast image using ImageJ software (NIH, Bethesda, MD, USA). Caspase-3/7 activity in these living differentiated RGC-5 cells under elevated pressure was probed using MR-(DEVD)_2_ (#936, Immunochemistry Technologies, LLC, Bloomington, MN). Lyophilized MR-(DEVD)_2_ was reconstituted with dimethyl sufloxide (DMSO) at the concentration of 3mM. The cells were treated with diluted MR-(DEVD)_2_/DMSO solution for 1 hour at 37ºC in the incubator with 5% CO_2_ at a concentration of 20 μM. Then, the cells were transferred into the pressurized incubation chamber residing on the fluorescence microscope for real-time imaging studies. The MR-(DEVD)_2_ is composed of the MR fluorophores (also known as cresyl violet in the literature) conjugated to two 4 amino acid sequences, aspartylglutamylalanylaspartic acid (DEVD). MR-(DEVD)_2_ can freely penetrate the cell membrane without cell lysis. When linked to two DEVD sequences, MR does not fluoresce. Following caspase induced enzymatic hydrolysis at one or both of the aspartic acid amide linkage sites, the MR fluorophores emit red (610-660 nm) photons when excited at 550-590 nm. Hence the fluorescence intensity from unit area of the cell increases with increased caspase-3/7 activation. Quantitative calibration of the functional relation between the MR fluorescence intensity and the caspase concentration has not been established given the difficulty in measuring the rate at which cleaved MR leaks from cellular space into the surrounding culture media. Here we use the fluorescence intensity from the caspase probe MR-(DEVD)_2_ loaded cells as the biomarker response without attempting to convert it into a concentration of activated caspases. Intracellular Ca^2+^ concentration is monitored using the Ca^2+^-sensitive dye Fluo-4 AM (F14217, Invitrogen, Merelbeke, Belgium). Upon binding of Ca^2+^ to Fluo-4, quantum yield of Fluo-4 increases from near 0 to 0.14 (at least 100 fold increase) while the emission wavelength is essentially unaffected (~520 nm, green). The cells for intracellular Ca^2+^ imaging were loaded with Fluo-4 AM at the concentration of 2μM in Fluo-4 loading medium of Hanks' Balanced Salt Solution (140 mM NaCl, 5 mM KCl, 4 mM NaHCO_3_, 0.3 mM Na_2_HPO_4_, 0.4 mM KH_2_PO_4_ , 6 mM D-Glucose without CaCl_2_, MgCl_2_, or phenol red) for 45 minutes at 37°C without adding any pluronic acid. The cells were washed with warm PBS to remove any dyes that were nonspecifically associated with the cell surface and incubated for a further 30 minutes to allow complete de-esterification of intracellular AM esters in imaging medium of phenol red-free growth medium of RGC-5. Then, the cells were transferred to the imaging pressurized chamber for live-cell imaging. After the dish was transferred into the imaging setup, it was first maintained at ~0 mmHg, 5%CO_2_, 37^o^C, and ~100% humidity. Then the pressure was elevated to 100 mmHg immediately before the start of imaging. For simultaneous imaging of intracellular Ca^2+^ and caspase-3/7, Fluo-4 AM and MR-(DEVD)_2_ were co-loaded by serially loading Fluo-4 AM first followed by MR-(DEVD)_2_ as stated above. No interfering effect of dyes during the co-loading was observed. Typically, for any given time point, phase-contrast images of cell morphology and fluorescent images of MR-(DEVD)_2_ (caspase-3/7) and Fluo-4 AM (Ca^2+^) were taken back-to-back. The area occupied by a cell determined from the phase-contrast image taken immediately (within 30 seconds) after a fluorescent image is used to define the ROI (region of interest) to calculate the area-normalized fluorescence intensity from the cell (total fluorescence intensity from the ROI divided by the area of the ROI).

## Statistical Analysis

Statistical analysis of the potential relation between the time of cell morphological change (cell body shrinkage time, *t*_shrk_, and the neurite retraction time, *t*_retr_) and the time of the two intracellular molecular processes (the time of Ca^2+^ peak, *t*_Ca2+_, and the time of caspase-3/7 activation, *t*_csp_) observed simultaneously in the population of 23 cells (data presented in Fig. 7 and Fig. S2) was carried out. The results are summarized in Table S1 and discussed below.

As shown in Fig. 7A and S2A, there are apparent outliers in the data of *t*_shrk_ vs. *t*_Ca2+_ and *t*_retr_ vs. *t*_Ca2+_. The outliers are suspected to originate from spontaneous morphological changes of cell body shrinkage and neurite retraction unrelated to the pressured-induced apoptosis. Indeed even in the four runs of control experiments at 15 mmHg, live RGC cells exhibit random morphological change and ~18% (a total of 14 cells out of 77 in four runs of control experiments under 15 mmHg: 3 out of 20 cells in caspase-3/7 and morphology imaging run, 4 out of 14 cells in the Ca^2+^ and morphology imaging run, and 7 out of 43 cells in two Ca^2+^, caspase-3/7, and morphology imaging runs) of the cells exhibited spontaneous cell body shrinkage and neurite retraction without observable Ca^2+^ peak and caspase-3/7 activation. Thus it can be anticipated that cell body shrinkage in a similar fraction (18%) of the 23 cells (~4 cells) under 100 mmHg shown in Fig. 7A and B and Fig. S2A and B might be spontaneous and not related to either Ca^2+^ or caspase-3/7. Therefore a check was deemed necessary to retain or exclude such “outlier” cells so that the true relation between the time of the morphological change and the Ca^2+^ and/or caspase-3/7 activation can be ascertained.

For such purposes, we assume that (*t*_shrk_ - *t*_Ca2+_),( *t*_shrk_ - *t*_csp_) , (*t*_retr_ - *t*_Ca2+_) , and (*t*_retr_ - *t*_csp_) in the cell population follow normal distribution. Then, guided by the observed behavior of the cells in the control experiment noted above, a maximum of four cells can be candidates for discarding as outliers. Applying the generalized extreme studentized deviate (ESD) test [S4,S5], we find that one data point in Fig. 7A and three data points in Fig. S2A should be rejected as “outliers” with 95% confidence level. These outliers are highlighted by red circles in Fig. 7A and Fig. S2A. No outlier can be rejected in Fig. 7B and Fig. S2B.

Table S1: Summary of the result of statistical analysis on the relation between the time of morphological changes (*t*shrk, *t*retr) and the time of intracellular molecular processes (*t*Ca2+, *_t_*_csp)_.

|  | Between *t*_shrk_  and *t*_Ca2+_ | Between *t*_shrk_ and *t*_csp_ | Between *t*_retr_ and *t*_Ca2+_ | Between *t*_retr_ and *t*_csp_ |
| --- | --- | --- | --- | --- |
| Pearson Correlation Coefficient (*r*) | 0.66 | 0.06 | 0.85 | 0.29 |
| Significance (*p*) of the correlation coefficient | 1E-3 | 0.78 | 2E-6 | 0.18 |
| Mean time difference (morphological change to molecular process) (hours) | 0.57 | -6.5 | 0.20 | -7.2 |
| Standard deviation (σ) of time difference (hours) | 1.7 | 4.0 | 0.84 | 3.3 |

After rejecting the outlier cells, we calculate that the time of cell body shrinkage and Ca^2+^ elevation (*t*_shrk_ and *t*_Ca2+_) are positively correlated with a Pearson correlation coefficient of 0.66 (significance *p*=1E-3)**.** In comparison, the correlation coefficient between *t*_shrk_ and *t*_csp_ is only 0.06 (significance *p*=0.78). Moreover, the mean time difference between the cell body shrinkage and the Ca^2+^ peak $\left\langle(t_{shrk}-t_{Ca2+})\right\rangle$ is 0.57h and the standard deviation is 1.7h. The mean time difference between the cell body shrinkage and the caspase-3/7 activation $\left\langle(t_{shrk}-t_{csp})\right\rangle$ is -6.5h and the standard deviation is 4.0h. Thus, on average, the caspase-3/7 activation occurs considerably later than the cell body shrinkage. (Hypothesis $\left\langle(t_{shrk}-t_{csp})\right\rangle\geq0$ is rejected by Student’s t-test with 99.99% confidence level.) Furthermore, the time difference between the cell body shrinkage and the Ca^2+^ peak occurrence is more narrowly distributed than the time difference between cell body shrinkage and caspase-3/7 activation (σ(*t*_shrk_ - *t*_Ca2+_)<σ(*t*_shrk_ - *t*_csp_), Student’s t-test confidence level 99.99%).

Similarly to the time of cell body shrinkage, the time of neurite retraction (*t*_retr_) is highly correlated with the time of Ca^2+^ elevation (*t*_Ca2+_). The Pearson correlation coefficient is 0.85 (significance *p*=2E-6). In comparison, the correlation coefficient between *t*_retr_ and *t*_csp_ is only 0.29 (significance *p*=0.18). Moreover, the mean time difference between the neurite retraction and the Ca^2+^ peak $\left\langle(t_{retr}-t_{Ca2+})\right\rangle$ is 0.20h and the standard deviation is 0.84h. In comparison, the mean time difference between the cell body shrinkage and the caspase-3/7 activation $\left\langle(t_{retr}-t_{csp})\right\rangle$ is -7.2h and the standard deviation is 3.3h. Thus on average the caspase-3/7 activation occurs later than the neurite. (Hypothesis$\left\langle(t_{retr}-t_{csp})\right\rangle\geq0$ is rejected by Student’s t-test with 99.99% confidence level.) Furthermore, the time difference between the neurite retraction and the Ca^2+^ peak is more narrowly distributed than the time difference between neurite retraction and caspase-3/7 activation (σ(*t*_retr_ - *t*_Ca2+_)<σ(*t*_retr_ - *t*_csp_), Student’s t-test confidence level 99.99%).

Summarizing the analysis above, we conclude that our simultaneous multiple-process imaging data provide statistically meaningful support for the view in the literature [S6] that the ion channel activity leading to water efflux from the cell is responsible for the early apoptotic morphological changes in RGC-5 under elevated hydrostatic pressure without the need for significant caspase-3/7 activation.

**References**

S1. Krishnamoorthy RR, Agarwal P, Prasanna G, Vopat K, Lambert W, et al. (2001) Characterization of a transformed rat retinal ganglion cell line. Brain Res Mol Brain Res 86: 1-12.

S2. Wong RG, Hadley RD, Kater SB, Hauser GC (1981) Neurite outgrowth in molluscan organ and cell cultures: the role of conditioning factor(s). J Neurosci 1: 1008-1021.

S3. Wong RG, Martel EC, Kater SB (1983) Conditioning Factor(S) Produced by Several Molluscan Species Promote Neurite Outgrowth in Cell-Culture. Journal of Experimental Biology 105: 389-393.

S4. Iglewicz B, Hoaglin DC (1993) How to Detect and Handle Outliers. Milwaukee, WI: ASQ Quality Press. pp. 32-33.

S5. Rosner B (2006) Fundamentals of Biostatistics. Belmont, CA: Duxbury Press. pp. 328-329.

S6. Maeno E, Ishizaki Y, Kanaseki T, Hazama A, Okada Y (2000) Normotonic cell shrinkage because of disordered volume regulation is an early prerequisite to apoptosis. Proc Natl Acad Sci U S A 97: 9487-9492.
